# Theorizing Cultural and Social Intersections of Age and Gender: The Approach of Anocriticism

**DOI:** 10.1093/geroni/igaa057.3016

**Published:** 2020-12-16

**Authors:** Roberta Maierhofer

**Affiliations:** University of Graz, Graz, Steiermark, Austria

## Abstract

This contribution discusses empirical applications of the approach of ‘anocriticism’ in interdisciplinary gerontological research. Despite the connection in terms of epistemology and ontology, the intersection of gender and age has been mostly ignored, privileging works focusing either on age or gender (Calasanti & Slevin 2001:27; Denninger & Schütze 2017:7). Age/ing Studies, however, would not have been established as a field without the theoretical and methodological approaches of feminist theory (Maierhofer (2019:2). Anocriticism was originally developed in order to investigate cultural representations of age/ing (Maierhofer 2003, 2004b, 2004a, 2007, 2012), but has recently been taken up in social sciences (Ratzenböck 2016a, 2016b, 2017a, 2017b; Gales and Loos 2020, forthcoming) in order to draw attention to four dimensions: (a) age and aging’s collective cultural construction and relation to gender, (b) the individual dimension of aging, (c) people’s interpretative power and narrative performance, and (d) age/ing’s potential for resistance and change.

